# Tumor-targeting hydroxyapatite nanoparticles for remodeling tumor immune microenvironment (TIME) by activating mitoDNA-pyroptosis pathway in cancer

**DOI:** 10.1186/s12951-023-02231-4

**Published:** 2023-12-07

**Authors:** Yuxuan Yang, Jia Yang, Nan Zhu, Haosen Qiu, Wenxiang Feng, Ying Chen, Xinhua Chen, Yuehong Chen, Wenbo Zheng, Min Liang, Tian Lin, Jiang Yu, Zhaoze Guo

**Affiliations:** 1 Department of General Surgery, Nanfang Hospital, Southern medical University, Guangzhou, 510515 China; 2 Department of General Surgery, Xiangyang Central Hospital, Affiliated Hospital of Hubei University of Arts and Science, Xiangyang, 441021 China; 3 Department of Oncology, Innovation Centre for Advanced Interdisciplinary Medicine, Guangzhou Key Laboratory of Enhanced Recovery after Abdominal Surgery, The Fifth Affiliated Hospital of Guangzhou Medical University, Guangzhou Medical University, Guangzhou, 510700 China; 4 Breast Division, Department of General Surgery, Nanfang Hospital, Southern Medical University, Guangzhou, 510515 China

**Keywords:** Tumor-targeting, OX-mitoDNA, Pyroptosis, HAP, TIME

## Abstract

In recent years, immunotherapy has emerged as a promising strategy for treating solid tumors, although its efficacy remains limited to a subset of patients. Transforming non-responsive “cold” tumor types into immuno-responsive “hot” ones is critical to enhance the efficacy of immune-based cancer treatments. Pyroptosis, a programmed cell death mechanism, not only effectively eliminates tumor cells but also triggers a potent inflammatory response to initiate anti-tumor immune activities. This sheds light on the potential of pyroptosis to sensitize tumors to immune therapy. Hence, it is urgent to explore and develop novel treatments (e.g., nanomedicines) which are capable of inducing pyroptosis. In this study, we constructed tumor-targeting nanoparticles (CS-HAP@ATO NPs) by loading atorvastatin (ATO) onto chondroitin sulfate (CS) modified hydroxyapatite (HAP) nanoparticles (CS-HAP). CS was strategically employed to target tumor cells, while HAP exhibited the capacity to release calcium ions (Ca^2+^) in response to the tumor microenvironment. Moreover, ATO disrupted the mitochondrial function, leading to intracellular energy depletion and consequential changes in mitochondrial membrane permeability, followed by the influx of Ca^2+^ into the cytoplasm and mitochondria. CS and HAP synergetically augmented mitochondrial calcium overload, inciting the production of substantial amount of reactive oxygen species (ROS) and the subsequent liberation of oxidized mitochondrial DNA (OX-mitoDNA). This intricate activation process promoted the assembly of inflammasomes, most notably the NLRP3 inflammasome, followed by triggering caspase-1 activation. The activated caspase-1 was able to induce gasderminD (GSDMD) protein cleavage and present the GSDM-N domain, which interacted with phospholipids in the cell membrane. Then, the cell membrane permeability was raised, cellular swelling was observed, and abundant cell contents and inflammatory mediators were released. Ultimately, this orchestrated sequence of events served to enhance the anti-tumor immunoresponse within the organism.

**Supplementary Information:**

The online version contains supplementary material available at 10.1186/s12951-023-02231-4.

## Introduction

Tumor immunotherapy stands as a strategic approach that harnesses the capabilities of the innate immune system to boost anti-tumor responses, leading to the effective elimination of malignant cells [1]. In 2013, tumor immunotherapy was valued as the top of ten scientific breakthroughs of the year, while the Nobel Prize in physiology or medicine in 2018 was awarded to advances in the same field [2]. Therefore, immunotherapy ushered in a paradigm shift, reshaping traditional treatment of cancer, leading the third revolution in oncology intervention after the era of conventional chemotherapy and targeted therapies [3, 4]. Given that only a small subset of patients can benefit from immunotherapy, how to elicit immune responses specifically against tumor antigens within the body and thus transform a substantial portion of non-responsive “cold” tumor types into responsive “hot” tumor types remained an essential issue to be addressed [5–7].

Pyroptosis represents a programmed cell death mechanism orchestrated by the Gasdermin (GSDM) protein family [8]. This process is characterized by distinct features, including DNA fragmentation, chromatin condensation, cellular swelling, and the formation of cellular bubbles, which are concomitant with the release of a vast amount of inflammatory molecules and intracellular contents [9, 10]. There are two principal activation pathways that underlie pyroptosis, including the cononical pathway, where inflammasomes activate caspase-1, and the non-cononical pathway, where cytosolic lipopolysaccharides (LPS) trigger the activation of caspase-4/5/11 [11, 12]. In the cononical pathway, the pivotal gasderminD (GSDMD) protein within the cell is cleaved by activated caspase-1, yielding two distinct domains: the C-domain (GSDM-C) and the N-domain (GSDM-N). Notably, the GSDM-N domain displays an affinity for membrane phospholipids and forms pores on the cell membrane, resulting in cellular swelling and bubble formation [13, 14]. As cellular swelling persists, the permeabilized membrane ultimately ruptures and releases an ample quantity of cellular contents and inflammatory mediators, including IL-18, NF-κB, IL-1β, ATP, HMGB1, etc [15]. Of note, IL-1β can stimulate dendritic cell (DC) maturation and monocyte activation. It also exerts a direct influence on antigen-specific cytotoxic CD8^+^ T cell responses, augments the number of Th1 CD4^+^ T cells, and suppresses the differentiation of immunosuppressive regulatory T cells [16–18]. Similarly, IL-18 can induce the production of gamma-interferon (γ-IFN) and recruit natural killer (NK) cells [19, 20]. As a result, pyroptosis has gained remarkable prominence as an innovative strategy in cancer treatment. In 2020, pyroptosis is further recognized for its potential in the elimination of malignant cells by triggering robust inflammatory response and anti-tumor immune activity in the body [21–23]. However, the existing small molecules or chemotherapy drugs related to pyroptosis face challenges such as blood circulation, non-specific biological distribution, and adverse reactions. Therefore, there is a pressing need to develop pyroptosis-inducers that are both safe and efficient.

It is universally known that Ca^2+^ stands as a key ion governing various important physiological activities such as signal transduction, energy metabolism, protein phosphorylation and dephosphorylation, cell proliferation and differentiation, and apoptosis [24]. Normally, the free and bound intracellular Ca^2+^ is regulated by both the mitochondria and the endoplasmic reticulum to maintain a dynamic balance [25, 26]. Research has demonstrated that an excessive influx of Ca^2+^ into cells has a detrimental impact on cell function. Elevated Ca^2+^ levels can result in heightened cellular oxidative stress, leading to the generation of a substantial quantity of reactive oxygen species (ROS). High levels of intracellular ROS actively target mitochondrial DNA (mitoDNA) and transform it into oxidized mitochondrial DNA (OX-mitoDNA). OX-mitoDNA can be repaired by the DNA glycosylase (OGG1) when the cells are functioning properly. Nevertheless, in cases of cellular dysfunction, OX-mitoDNA is cleaved into fragments measuring 500–650 bp by the Flap Endonuclease 1 (FEN1). These fragments, when released into the cytoplasm through the open mitochondrial permeabilization transition pore (mPTP), are then recognized and bound by NLRP3. [27–30]. Presently, several Ca^2+^ overload nano-inducers have demonstrated efficacy in stimulating immunogenic cell death (ICD) mediated by mitochondrial Ca^2+^ overload [31]. Hydroxyapatite (HAP), a biomaterial analogous to the inorganic components of human hard tissues such as bones and teeth, possesses exceptional biocompatibility [32]. Our previous research has demonstrated that HAP-released Ca^2+^ could selectively harms tumor cells by inducing mitochondrial Ca^2+^-related damage [33]. Therefore, nanoscale HAP is an optimal Ca^2+^ overload nano-inducer. Nonetheless, using solely HAP nanoparticles would not achieve tumor-specific targeting, and the amount of Ca^2+^ they release exerts limited mitochondrial cytotoxicity, making it challenging to induce effective mitochondrial DNA (mitoDNA) damage and a robust inflammatory pyroptotic immune response. Chondroitin sulfate (CS), a polysaccharide composed of disaccharide units of β-1,3-N-acetylglucosamine and β-1,4-D-glucuronic acid, is abundantly present in human arteries, cartilage, and ligaments [34]. CS is a hydrophilic group that exhibits favorable biocompatibility and degradability, and it plays a role in promoting cell proliferation, exerting anti-thrombotic and anti-inflammatory effects, as well as targeting specific cells. For instance, some tumor cells exhibit a significant presence of CD44v receptors on their cell membranes. The CD44v receptor, a variant of the CD44 receptor, serves as a specific binding site for hyaluronic acid and enables cells to specifically recognize and interact with glycosaminoglycan-based biomaterials like CS [35, 36]. Furthermore, atorvastatin (ATO), usually an enzyme inhibitor, wields negative effects on the mevalonate pathway by competitively inhibiting HMG-CoA reductase activity and curtails the production of cellular ubiquinone (CoQ10) [37]. Depletion of CoQ10, an essential component of the mitochondrial electron transport chain, triggers malfunction in mitochondrial respiratory chain, leading to disruptions in energy supply and alterations in the permeability of the mitochondrial outer membrane, followed by an influx of cytoplasmic Ca^2+^, which culminates in mitochondrial Ca^2+^ overload [38, 39]. Therefore, the combination of ATO on CS-modified HAP is supposed to be an efficient tumor-targeting nanoparticles for activating the OX-mitoDNA-pyroptosis pathway, which has rarely been reported before.

**Scheme 1:**
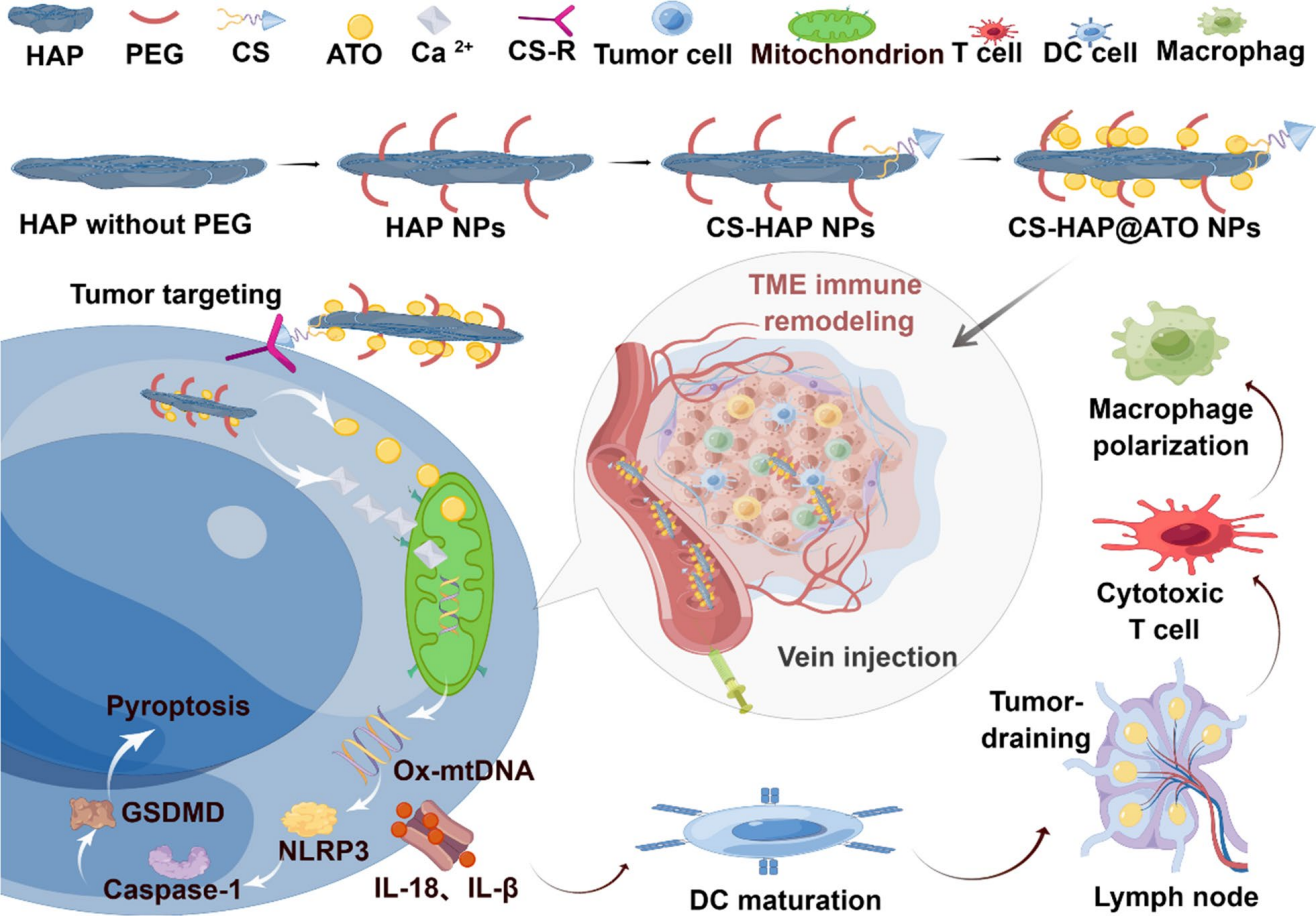
Schematic illustration of tumor-targeting hydroxyapatite nanoparticles for remodeling tumor immune microenvironment (TIME) by activating OX-mitoDNA-pyroptosis pathway in colorectal cancer

In our study, we constructed tumor-targeting nanoparticles (CS-HAP@ATO NPs) by loading ATO onto CS modified HAP nanoparticles. From the mechanism diagram (Scheme 1), CS-HAP@ATO employs CS to selectively target tumor cells; HAP responsively releases Ca^2+^ when exposed to tumor microenvironment; ATO simutaneously hampers mitochondrial function, resulting in energy disturbance and permeablization of mitochondrial outer membrane; these cumulative effects elicit cytoplasmic Ca^2+^ influx into mitochondria. Then, considerable amounts of ROS is generated and OX-mitoDNA is liberated. Such cascade prompts the assembly of NLRP3 inflammasome, followed by triggering caspase-1 activation. The activated caspase-1 is able to induce gasderminD (GSDMD) protein cleavage and present the GSDM-N domain, which interacts with phospholipids in the cell membrane. Next, pores are formed on the cell membrane and cellular swelling was observed. This complex process induces the release of substantial cellular contents and inflammatory mediators, and ultimately enhances the anti-tumor immunoresponse within the organism.

## Materials and methods

### Materials

Hydroxyapatite (HAP, particle size 80 nm), chondroitin sulfate (CS), and atorvastatin (ATO) were procured from Shanghai Macklin Biochemical Technology Co., Ltd. Methoxy PEG silane (Mw = 2000, 5 mg) was sourced from Guangzhou Carbonhydrate BioTech Co., Ltd. N-Hydroxysuccinimide (NHS) and 1-(3-Dimethylaminopropyl)-3-ethylcarbodiimide hydrochloride (EDC) were obtained from Shanghai Aladdin Reagent Co., Ltd. The CCK-8 assay kit was provided by Shanghai Beyotime Biotechnology. The EDU cell proliferation assay kit was purchased from Dalian Meilun Biotechnology Co., Ltd. The JC-1 enhanced mitochondrial membrane potential detection kit was acquired from Shanghai Beyotime Biotechnology. The mitochondrial reactive oxygen species assay kit (MitoROS) was sourced from Wuhan Aimijie Technology Co., Ltd. The mitochondrial permeability transition pore detection kit (MPTP) was obtained from Shanghai Beibo Biotechnology Co., Ltd. Caspase-1 (caspase-1 and cleaved caspase-1), GSDMD NT, NLRP3, and WB lysis buffer were acquired from Affinity Biosciences, USA. Phosphate-buffered saline (PBS), fetal bovine serum (FBS), 0.05% trypsin, and DMEM culture medium were purchased from Gibco, USA. Four-week-old female BALB/c mice were obtained from the Animal Experimental Center of Guangdong Provincial Center for Experimental Animals.

### Synthesis of CS-HAP@ATO NPs

Initially, HAP (25 mg) and methoxy polyethylene glycol (PEG) silane (125 mg) were dissolved in 25 ml of anhydrous ethanol. Subsequently, consistent agitation was upheld using a magnetic stirrer at 60 °C for 24 h. The resultant mixture underwent multiple washes with anhydrous ethanol and deionized water to obtain purified HAP NPs. APTES (100 mg) was dissolved into 30 ml of anhydrous ethanol through vigorous stirring for 2 h, and HAP NPs (100 mg) were then dispersed into the mixture. Stirring ensued for an additional 3 h, followed by adjusting the pH to 9–10 with aqueous ammonia. The mixture underwent multiple cleansing steps using anhydrous ethanol and deionized water. Subsequently, HAP-NH_2_ NPs were obtained using vacuum drying. CS (20 mg) was dissolved in 20 ml of deionized water to yield a CS solution. To activate the carboxyl groups on CS, EDC (80 mg) and NHS (11.7 mg) were then added into CS solution. The solution was then subjected to stirring over 1–3 h. Next, the pH was adjusted to a range of 6–7 using hydrochloric acid. The carboxyl group in CS is completely activated by EDC and forms a stable intermediate with NHS. 25 mg of HAP-NH_2_ NPs were incorporated under stirring conditions while the mixture was agitated at room temperature for 24 h. The composite was then subjected to dialysis using a membrane characterized by a molecular weight cut-off (MWCO) value of 3500 Da, with deionized water as the medium for 48 h. CS-HAP NPs were acquired after freeze-drying. Following that, a blend of CS-HAP NPs (25 mg) and atorvastatin (ATO) (25 mg) was dissolved into 25 ml of deionized water. The resultant mixture was subjected to stirring at room temperature for 24 h. After dialysis using a membrane of MWCO value of 3500 Da amid deionized water over 48 h and freeze-drying in a vacuum dryer, CS-HAP@ATO NPs were finally acquired for later research.

### Synthesis of FITC labelled HAP-based NPs

To graft FITC onto HAP NPs, we first covalently linked 1 mg FITC to 10 µL APTMS in 1 mL ethanol in the dark for 12 h. Then, 100 mg HAP-based NPs were dispersed in 10 mL ethanol, and mixed with 1 mL FITC/APTMS ethanol solution (1 mg/mL). After stirring under dark conditions for 24 h, the HAP-based NPs were centrifuged and washed with ethanol until the supernatants were colorless, and then CS was labelled with HAP-based NPs by an amidation reaction.

### Structural characterization of CS-HAP@ATO NPs

The surface morphology of CS-HAP@ATO NPs was observed through transmission electron microscopy (TEM; JEM-2100 F, JEOL, Japan). Structural elucidation and elemental profiling of CS-HAP@ATO NPs were performed using elemental mapping images, X-ray diffraction (XRD; Ultima IV, Rigaku, Japan), X-ray photoelectron spectroscopy (XPS;Thermo Fisher K-Alpha, Thermo Fisher Scientific, USA), Fourier-transform infrared spectroscopy (FT-IR; Nicolet IS5, Thermo Fisher Scientific), and ultraviolet-visible spectrophotometry (UV–Vis; UV-2600, Shimadzu, Japan).Thermogravimetric analysis (NETZSCH STA 449F3, Germany) was used to detect the thermal stability of the synthesized products. The drug loading ratio and encapsulation efficiency were then calculated. The Zeta potential and particle size of the synthesized products were gauged using a Zetasizer Nano-ZS (Malvern).

### pH-responsive drug release of CS-HAP@ATO NPs

We configured the CS-HAP@ATO NPs with phosphate-buffered saline (PBS) to 200 µg/mL. This solution was portioned into four samples, with the pH levels adjusted to 6.0, 6.8, 7.0 and 7.4, respectively. Under controlled conditions, these mixtures underwent stirring at 37 °C at 50 rpm. A volume of 3.0 mL of the release medium was harvested and replaced with fresh medium at predetermined time intervals. The ATO concentrations in the harvested samples were determined through UV spectrophotometer. Similarly, the calcium ion (Ca^2+^) content was quantified utilizing a calcium assay kit (C004-2-1, Nanjing Jiancheng Bioengineering Institute).

### Cell culture

Mouse colorectal cancer (CRC) cells, called CT26, were purchased from the American Type Culture 160 Collection and cultured in Dulbecco’s Modified Eagle Medium (DMEM), supplemented with 10% Fetal Bovine Serum (FBS), 100 IU/mL penicillin, and 100 µg/mL streptomycin. These cells were incubated at 37 °C in an incubator, maintaining CO_2_ concentration at 5%. When the cells have covered about 80% of the bottom area of the culture bottle, they are trypsinized and inoculated into cell culture dishes or multiwell plates for passage or analysis.

### Cellular uptake detection

CT26 cells were inoculated in confocal culture dishes with a density of 5 × 10^3^ per well and cultured for 24 h. CT26 cells were then incubated with CS-HAP@ATO NPs and HAP@ATO NPs labeled with fluorescein isothiocyanate (FITC), respectively. After incubating with CS-HAP@ATO NPs for 8 h, the intracellular ultrastructure was observed by TEM, or fixed with 4% paraformaldehyde (PFA) after incubation, stained with 4’, 6-diaminidine 2-phenylindole (DAPI) for 8 min, finally observed by confocal fluorescence microscopy (CLSM).

### Cell viability assays

For the cytotoxicity of synthetic NPs, CT26 cells were seeded in 96-well plates and cultured for 12 h. After 24 h of incubation with different concentrations of HAP NPs, HA (HAP@ATO NPs), CH (CS-HAP NPTs), or CHA (CS-HAP@ATO NPs), cell absorbance was determined in a microplate reader using the Cell Counting Kit-8 (CCK8). Furthermore, treated-CT26 cells were also detected using the live/dead cell staining kit and the 5-ethynyl-2’-deoxyuridine (EDU) cell proliferation assay kit. Meanwhile, treated CT26 cells were also observed by scanning clectron microscopy (SEM).

### Mitochondrial damage assessment

After incubating the CT26 cells with HAP, HA, CH, or CHA for 24 h, mitochondrial function was evaluated using enhanced mitochondrial membrane potential (MMP) assay kit (JC-1, provided by Shanghai Biyuntian Biotechnology), mitochondrial reactive oxygen species assay kit (MitoROS, provided by Wuhan Aimijie Technology), and mitochondrial permeability transition pore assay kit (MPTP, provided by Shanghai Beibo Biotechnology). These assays were employed to assess mitochondrial membrane potential, reactive oxygen species levels, and permeability transition pore activity.

### Oxidized mitochondrial DNA (Ox-mitoDNA) detection

CT26 cells were seeded into confocal dishes and subjected to a 24-h treatment with the mentioned samples. Following treatment, the cells were fixed with a 4% PFA solution, permeabilized using 0.1% Triton-100, and subsequently blocked utilizing a 5% BSA solution. After blocking, the cells were subjected to an overnight incubation at 4 °C with a primary antibody specifically targeting 8-hydroxy-2’-deoxyguanosine (8-OHdG) (bs-1278R, Bioss USA), which is indicative of oxidized mitochondrial DNA (Ox-mitoDNA). Washed by PBS three times, the cells were then treated with a secondary antibody against the primary antibody for 60 min. Ultimately, CT26 cells were stained with DAPI and observed under CLSM to quantify the levels of intracellular Ox-mitoDNA.

### Intracellular Ca^2+^ release

To investigate the intracellular Ca^2+^ release, CT26 cells were incubated with all samples (HAP NPs, HA, CH or CHA, respectively) for 24 h. The intracellular calcium ion density in each group was detected using the Fluo-4 AM calcium ion fluorescent probe.

### Western blot analysis

CT26 cells were inoculated into a 6-well plate and allowed to proliferate for 12 h. Subsequently, (HAP NPs, HA, CH or CHA were added. After 24 h, cells were harvested and thoroughly washed with PBS. Cell pellets were added into RIPA lysis buffer (provided by Shanghai Biyuntian Biotechnology) and the lysates was centrifuged to extract protein. The protein concentration was determined by the BCA protein assay kit. Next, protein electrophoresis was carried out, followed by transference of the proteins onto a nitrocellulose membrane. Subsequently, the membrane underwent blocking using a 5% BSA solution for 1 h at room temperature. After this, the membrane was incubated overnight at 4 °C with specific antibodies against various target proteins. The membrane was then washed with PBS and treated with appropriate anti-rabbit or anti-mouse IgG antibodies for 1 h at room temperature. As for detection and quantification of protein levels, including NLRP3, caspase-1, cleaved caspase-1, and GSDMD NT, were performed using Compass software.

### Caspase-1 fluorescence assay

Following a 24-hour incubation of cultured CT26 cells with HAP NPs, HA, CH, or CHA in 96-well plates, the intracellular content of caspase-1 was assessed using the FAM-FLICA caspase assay kit (supplied by Aimijie Technology, USA). The FAM-FLICA working solution was prepared according to the manufacturer’s instructions and then added into the wells. After incubation, cells were examined under a fluorescence microscope, and images were captured for quantitative analysis.

### The hemolytic test

A blood sample of 3 mL was subjected to centrifugation at 3000 rpm for 15 min at a temperature of 4 ℃, and the supernatant (white blood cells and plasma) was discarded. The red blood cells were washed through suspension and centrifugation for 1–2 cycles (3000 rpm, 15 min, 4 ℃) until the supernatant was colourless. The sedimented cells were resuspended to prepare a 2% erythrocyte suspension using PBS. The cells were treated with distilled water as the positive control group and PBS as the negative control group. CS-HAP@ATO NPs solutions at various concentrations (7.5, 15, 30, 60, 120, 240, 480 µg/mL) were used as experimental group. The treated samples were incubated in a water bath at 37 ℃ for 4 h. All samples were centrifuged (3000 rpm, 15 min) and arranged in a linear fashion to capture pictures, and the supernatant was preserved. The absorbance at 450 nm of the supernatant of different groups was measured using a microplate reader (with 96-well plate). Hemolysis rate (%) = (OD (sample) -OD (PBS))/(OD (ddH_2_0) -OD (PBS)) *100%.

### In vivo antitumor evaluation

To comprehensively assess the potential antitumor effects of CS-HAP@ATO NPs, we established a subcutaneous tumor model using CT26 tumors in male BALB/c mice. The mice received subcutaneous injections of CT26 cells on their dorsal skin, allowing tumor growth until reaching a volume of 100 mm^3^. Subsequently, the mice were categorized into five groups. On days 0, 4, 8, and 12, each group received different treatments via tail vein injection: saline, HAP NPs, HA, CH, or CHA. Starting from the initial injection day until the conclusion of the experiment, we recorded both tumor volumes and mouse weights. On day 15, the mice were humanely euthanized, and the tumors were measured, weighed, and preserved in 4% paraformaldehyde for subsequent analysis. By implementing this experimental setup, we were able to gauge the in vivo efficacy of CS-HAP@ATO NPs in inhibiting tumor growth and inducing potential therapeutic effects. Monitoring parameters such as tumor volume, weight fluctuations, and various tissue analyses facilitated a comprehensive evaluation of the antitumor potential of the synthesized nanomedicine in a live animal model.

### Hematoxylin and eosin (H&E) staining and immunohistochemical (IHC) analysis

Tissues were fixed in 4% PFA, embedded, sectioned, and stained with H&E for microscopy. For IHC staining, sections were dewaxed, processed for antigen retrieval, quenched to inhibit endogenous peroxidase activity, and subsequently incubated with appropriate primary antibodies. The sections were then incubated with anti-mouse IgG for 30 min at 25 °C, rinsed with Tris-buffered saline, and incubated in 3,3-diaminobenzidine solution at 25 °C for 10 min. Finally, the sections were counterstained with hematoxylin.

### Enzyme-linked immunosorbent assay (ELISA) analysis

Mice bearing tumor xenografts are treated and sacrificed at the end of the experiment, after which tumor tissue is collected. The tumor tissues or treated cells were suspended in PBS, homogenized, and centrifuged at 12,000 rpm for 10 min. The ELISA kit was used to detect the expression levels of IL-18 and IL-1β in the detected tissues for each target. By assessing the cytokine expression in this manner, we were able to gain insights into the immune response and potential inflammatory activity within the tumor microenvironment (TME).

### In vivo analysis of tumor immune cell recruitment

To evaluate the impact of different treatments on immune cell populations, the BALB/c mouse subcutaneous CT26 tumor model was again adopted. Following the administration of various samples, tumor and spleen tissues were collected from the mice. Single-cell suspensions were prepared through digestion and filtration. To prevent non-specific binding, anti-mouse CD16/CD32 antibodies were applied for 15 min. Cells were then stained with eBioscience™ Fixable Viability Dye eFluor™ 506 for an additional 15 min to identify viable cells. For the assessment of dendritic cell (DC) maturation in vivo, cell labeling was carried out using Percp CY7-anti-CD11c, PE-anti-CD80, and APC-anti-CD86 antibodies. Flow cytometry was employed to detect and quantify the levels of mature DCs. The proportions of CD4 + and CD8 + T cells were determined using APC-anti-CD3, FITC-anti-CD4, and PreCP-Cy5.5-anti-CD8 antibodies. Moreover, the changes in the distribution of CD4 + and CD8 + T cells resulting from various treatments were also analyzed. To assess the presence of regulatory T (Treg) cells, FITC-anti-CD4 and PE-anti-Foxp3 antibodies were applied for cell processing. Proportional changes in Treg cells within the tissues were evaluated. Additionally, the analysis of macrophage subtypes was conducted using Percp CY5.5-anti-CD11b, APC-anti-CD86, and BV421-anti-CD206 antibodies. Flow cytometry allowed for the determination of the proportions of M1 and M2 macrophages within the tissues and their respective alterations in response to different treatments. This comprehensive immune cell analysis provided insights into the effects of the developed nanomedicines on immune cell populations within the tumor microenvironment and spleen.

### Statistical analysis

The statistical analysis outcomes are expressed as mean ± standard deviation (SD) derived from a minimum of three independent experimental repetitions. Significant variations were assessed using one-way or two-way analysis of variance (ANOVA). Statistical significance was determined utilizing SPSS 22.0 software, with a P-value below 0.05 regarded as indicative of statistically significant differences.

## Results and discussion

### Synthesis and characterization of CS-HAP@ATO NPs

**Fig. 1:**
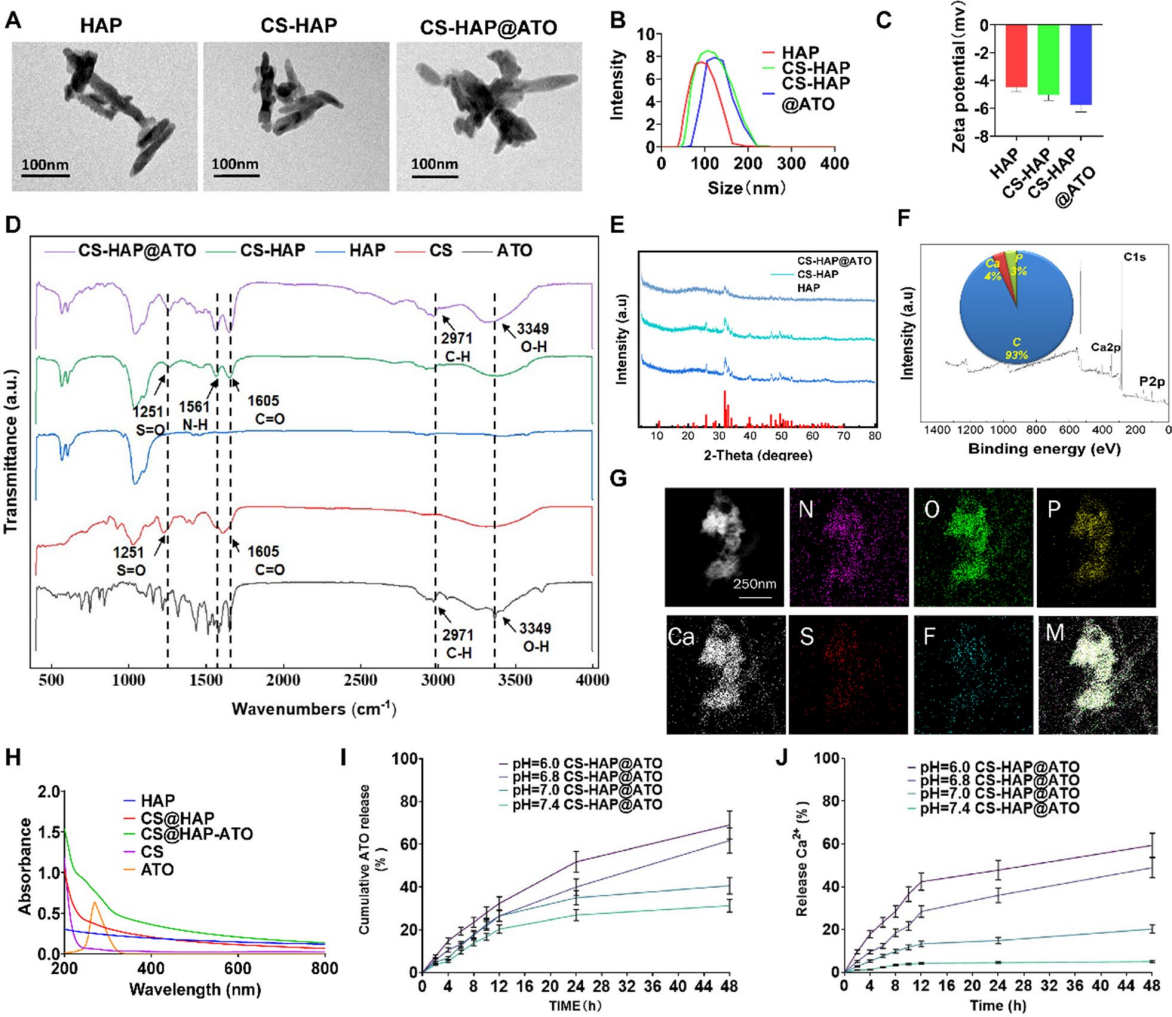
Structural characterization of CS-HAP@ATP NPs. **A** TEM images of HAP, CS-HAP and CS-HAP@ATO NPs. **B** DLS distribution of HAP, CS-HAP and CS-HAP@ATO NPs. **C** Zeta potentials of HAP, CS-HAP and CS-HAP@ATO NPs. **D** FT-IR spectra of HAP, CS, ATO, CS-HAP and CS-HAP@ATO. **E** XRD analysis of HAP, CS-HAP and CS-HAP@ATO NPs. **F** XPS analysis of CS-HAP@ATO NPs. **G** Elemental mapping images of CS-HAP@ATO NPs. **H** UV-is analysis of HAP, CS-HAP and CS-HAP@ATO NPs. **I** ATO release curve of HAP@ATO and CS-HAP@ATO NPs, at different pH conditions. **J** Ca^2+^ release curve of HAP@ATO and CS-HAP@ATO NPs, at different pH conditions. n = 3

TEM images illustrated that HAP, CS-HAP, and CS-HAP@ATO NPs displayed a rod-like structure with an approximate length of 100 nm (Fig. 1A). Dynamic light scattering (DLS) results indicated a size of around 100 nm for HAP, CS-HAP, and CS-HAP@ATO NPs (Fig. 1B). The zeta-potential measurements revealed values of -4 mV, -5 mV, and − 6 mV for HAP, CS-HAP, and CS-HAP@ATO NPs, respectively (Fig. 1C), suggesting minimal alteration in average zeta potential due to drug loading. To date, HAP has a positively charged on the surface and ATO has a negative charge, they were supposed to combine with each other through potential, which resulting into the increase of negative potential of our synthetic CS-HAP product. FT-IR spectroscopy outcomes aligned with previous findings, displaying characteristic peaks of HAP within the 600–1100 cm^−1^ range, including sharp peaks at 1037.58 cm^−1^ and 565.62 cm^−1^ attributed to PO4^3−^ group vibrations (Fig. 1D). Furthermore, S = O and carbonyl C = O stretching vibration peaks at 1251.575 m^−1^ and 1605.499 cm^−1^, which belong to CS, were observed in the CS-HAP spectrum. Interestingly, N-H bending vibration peak (the amide II band) was shown at 1561.567 cm^−1^, which was considered to be the amide bond generated by the binding of HAP and CS. Compared to CS-HAP group, O-H and C-H stretching vibration peaks of ATO appear at 3349.256 cm^−1^ and 2971.286 cm^−1^ appeared in CS-HAP@ATO NPs, indicating successful loading of ATO. XRD analysis corroborated the crystalline nature of HAP, CS-HAP, and CS-HAP@ATO NPs, with single crystalline phases observed in the materials (Fig. 1E). XPS results confirmed the presence of C, O, P, and Ca elements, with Ca 2p peaks at 347.13 eV, C 1s peaks at 284.95 eV, O 1s peaks at 531.99 eV, and P 2p peaks at 133.13 eV (Fig. 1F). The elemental composition of CS-HAP NPs encompassed 4% Ca, 30% P, and 93% C. Elemental mapping results indicated that CS-HAP@ATO NPs were mainly composed of Ca, N, O, P, S, and F (Fig. 1G). Specifically, S belongs to CS and F belongs to ATO, providing evidence for the successful formation of CS-HAP@ATO NPs. The thermogravimetric analysis results demonstrated that both HAP and CS-HAP@ATO NPs exhibited stability within the temperature range of 0 ℃ to 100 ℃, and they remained stable under common conditions. However, a significant drop in the mass of CS-HAP@ATO NPs was observed within 100 ℃ to 800 ℃, suggesting that CS-HAP@ATO NPs were less stable than HAP at this temperature range (Additional file 1: Fig. S1). UV-Vis spectroscopy showed distinct peaks at 275 nm for both the ATO and CS-HAP@ATO groups. The encapsulation efficacy (EE) and loading efficacy (LE) of ATO were determined to be 96% and 34%, respectively. The incorporation of CS introduced new peaks (190–200 nm) within the UV-Vis range, with CS content in CS-HAP NPs calculated as 41% (Fig. 1G). Collectively, these findings validated the successful fabrication of the CS-HAP@ATO nanocomposite.Prior research indicated that HAP could dissolve its deposited layer considerably when exposed to an acidic environment, signifying its potential for biodegradation in low-pH conditions, such as the TME. This property suggests the capacity of HAP to release both calcium and loaded drugs in response to pH variations. Thus, we investigated the pH-sensitive drug release profiles of ATO and Ca^2+^ in vitro. As depicted in Fig. 1I, J, at a neutral pH of 7.4, only about 30% of ATO and 5% of Ca^2+^ were released within 48 h. At a pH of 7.0, only about 40% of ATO and 20% of Ca^2+^ are released within 48 h. Contrastingly, under pH 6.8 conditions, the release of ATO and Ca^2+^ escalated to approximately 55% and 45%, respectively. Furthermore, in the pH 6.0 environment, the release rates accelerated to approximately 63% for ATO and 60% for Ca^2+^. These findings indicate the potential of CS-HAP@ATO NPs to exhibit enhanced drug release in the tumor microenvironment, which typically has a weakly acidic extracellular pH (ranging from pH 6.0 to 6.8).

### In vitro cellular uptake and anti-tumor effects of CS-HAP@ATO NPs

**Fig. 2:**
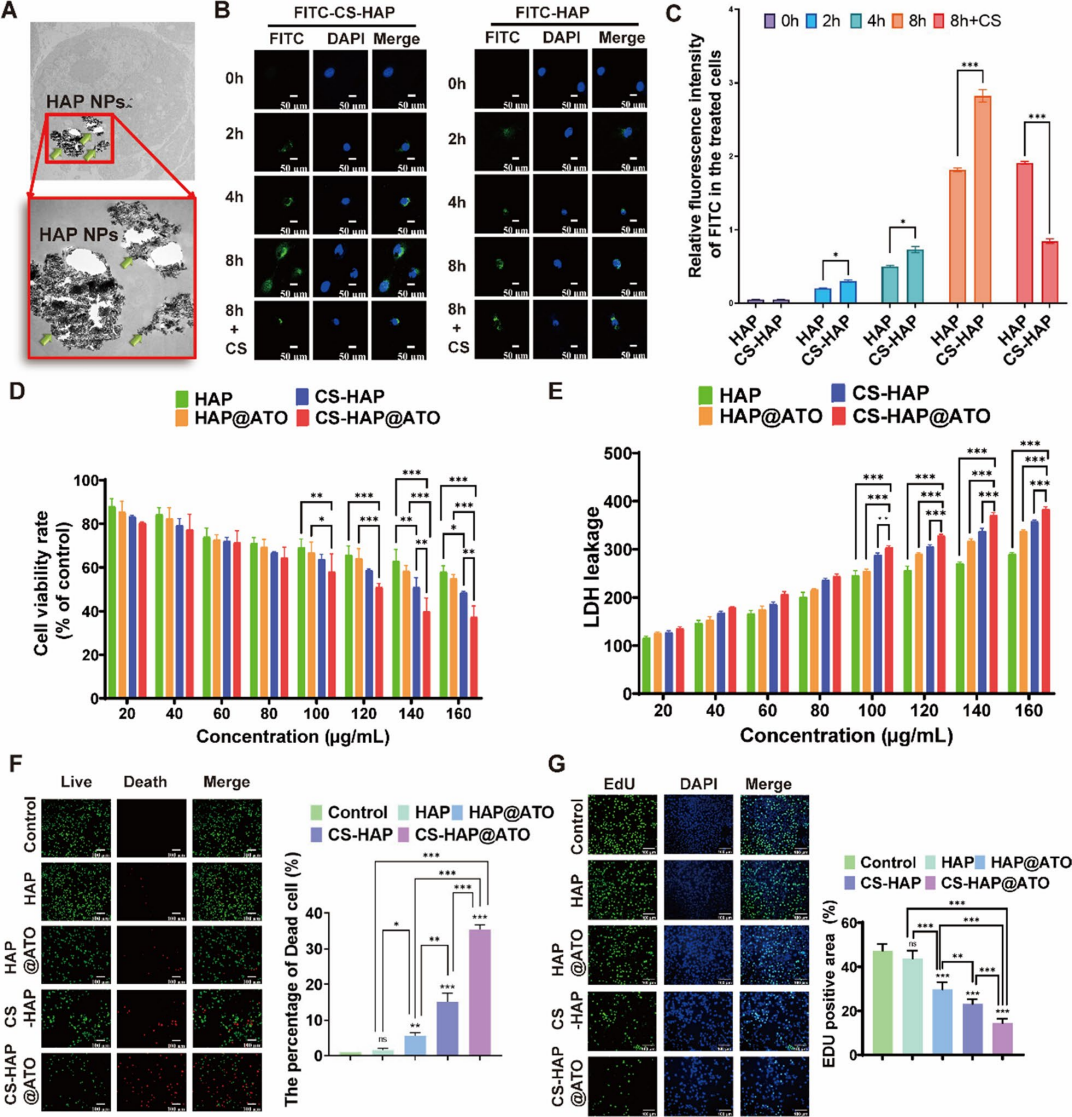
Cellular uptake and *in-vitro* antitumor effect of CS-HAP@ATO NPs. **A** TEM images of CT26 cells treated with CS-HAP@ATO NPs. **B**, **C** CLSM images of CT26 cells treated with FITC-labeled HAP@ATO and CS-HAP@ATO NPs (left) and fluorescence statistics inside CT26 cells, scale bar: 50 μm. **D** Cell viability of CT26 cells treated with various concentrations of HAP-based NPs. **E** LDH leakage of CT26 cells treated with various concentrations of HAP-based NPs at different concentrations. **F** Representative live/dead stained images (left) and quantitative live/dead results of CT26 cells after incubation with HAP-based NPs (cells in green are alive and cells in red are dead), scale bar: 100 μm. **G** Representative EdU stained images (left) and statistics EdU-positive rates of CT26 cells after incubation with HAP-based NPs, scale bar: 100 μm. n = 3. *P < 0.05, **P < 0.01, ***P < 0.001

The field of nanotechnology-based drug delivery systems has witnessed considerable growth as a promising alternative to conventional chemotherapy, largely due to their efficient drug delivery capabilities. In our previous studies, HAP demonstrated its potential as an innovative nanocarrier material for cancer-targeting NPs [33]. Nonetheless, the HAP NPs exhibited a limitation in selectively binding to cancer cells, which is a critical aspect that warrants further investigation. To address this limitation, we explored the potential of enhancing cancer cell targeting by modifying HAP NPs with CS. CD44, a cell surface receptor highly expressed on CRC cells, became our focus due to its potential significance. CS, known to specifically target the CD44 receptor, was selected as the modifying agent to facilitate the binding of CS-HAP NPs to CRC cells by recognizing this receptor on the tumor cell membrane [35]. In our study, CRC cells were incubated with CS-HAP@ATO NPs for 8 h, and observations were made using TEM. The TEM images (Fig. 2A) displayed the presence of numerous HAP NPs within the CRC cells. To visually monitor cellular uptake, CS-HAP@ATO NPs were labeled with FITC (green), and confocal laser scanning microscopy (CLSM) was employed to track the fluorescence intensity of FITC. The CLSM images (Fig. 2B, C) showcased a considerable increase in FITC intensity for CS-HAP@ATO NPs compared to the HAP@ATO group at various time points (2 h, 4 h, and 8 h). To ascertain the dependence of this uptake on CD44 receptors, we introduced free CS as a potential competitor for binding to CD44 receptors. Significantly, the introduction of free CS had a notable inhibitory effect on the cellular uptake of CS-HAP@ATO NPs, while no noticeable alteration was observed in the HAP@ATO group.

To comprehensively evaluate the antitumor effects of our developed NPs, we conducted a series of assays using CT26 cells. These assays aimed to elucidate the impact of different treatments on cell viability, cell death induction, and proliferation inhibition. Utilizing CCK-8 assays, we gauged the viability of CT26 cells after exposure to varying concentrations of HAP, CS-HAP, HAP@ATO, and CS-HAP@ATO solutions. Figure 2D demonstrates a significant reduction in cell survival rates across all groups with increasing concentrations. Notably, CS-HAP@ATO NPs exhibited the most pronounced anticancer effects among the treatments. Correspondingly, LDH leakage assays displayed a concentration-dependent increase in LDH release from CRC cells treated with HAP, CS-HAP, HAP@ATO, and CS-HAP@ATO NPs (Fig. 2E). Importantly, CS-HAP@ATO NPs induced the most substantial LDH leakage, surpassing the effects of the other treatments. Further substantiating our findings, Live/Dead assays were conducted using calcein-AM and PI staining. The results (Fig. 2F) revealed that the proportion of dead cells in the CS-HAP@ATO group was notably higher (25%) compared to the HAP (3%), CS-HAP (15%), and HAP@ATO (10%) groups. Importantly, the CS-HAP@ATO-treated group showed at least a 1.5-fold and 2-fold increase in dead cells compared to the HAP@ATO and CS-HAP groups, underscoring the superior efficiency of CS-HAP@ATO NPs in inducing cell death. We further evaluated the impact on cell proliferation using EdU, which incorporates into replicating DNA. The results (Fig. 2G) demonstrated that the CS-HAP@ATO-treated group exhibited the lowest percentage of EdU-positive cells (18%) compared to the control (50%), HAP (45%), HAP@ATO (30%), and CS-HAP (20%) groups. Collectively, our findings underscored that CS-HAP@ATO NPs effectively curbed tumor cell proliferation, largely attributed to the augmented CD44-receptor-mediated endocytosis pathway. While the enhanced antitumor effects were evident, the underlying intricate mechanism of CS-HAP@ATO NPs demands further elucidation.

### CS-HAP@ATO NPs caused mitoDNA damage

**Fig. 3:**
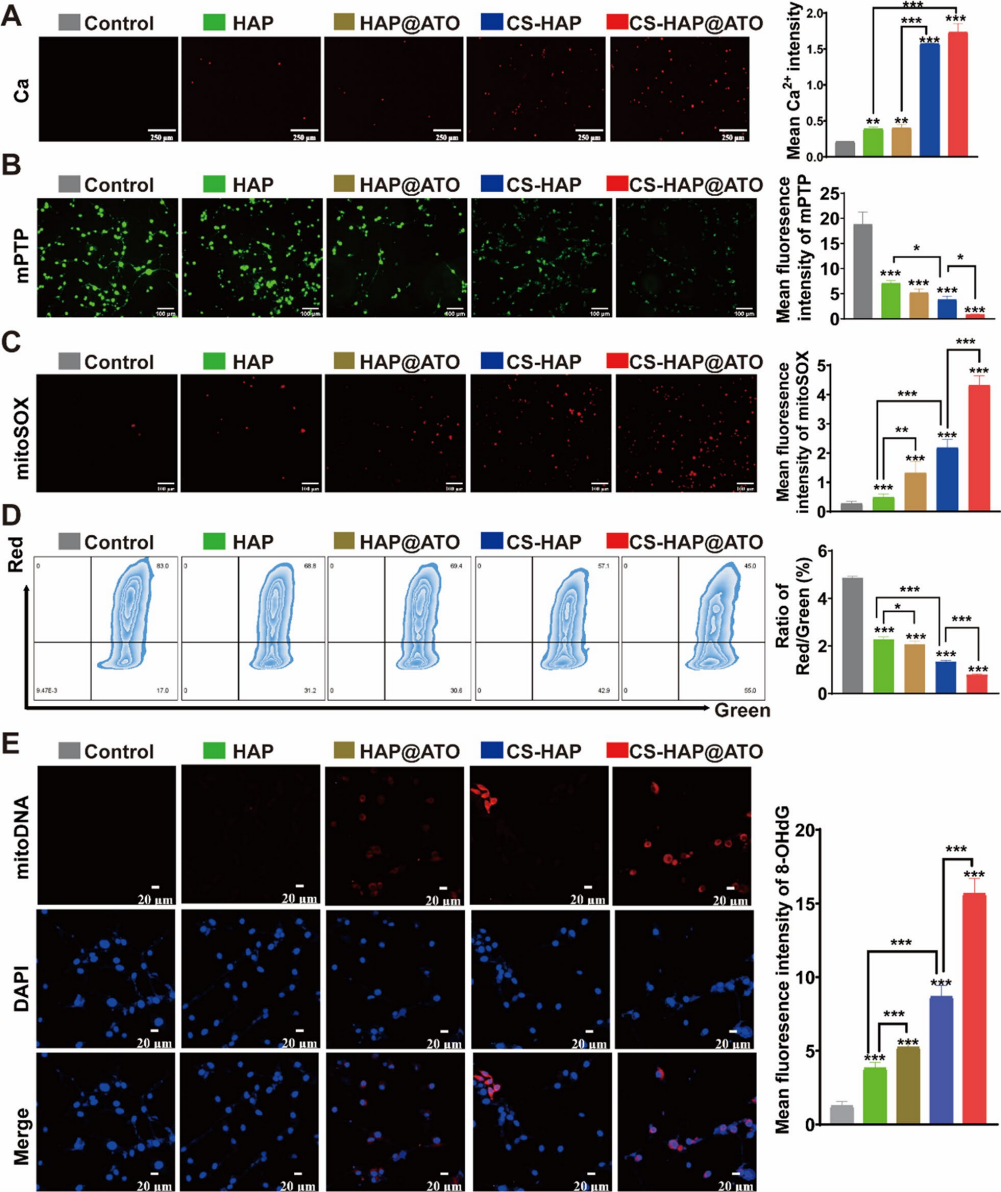
Dual mitoDNA damage properties of CS-HAP@ATO NPs. **A** CLSM images of Ca^2+^ (green) in CT26 cells (left), and the statistic green intensity (right). Scale bar: 100 μm. **B** mPTP images of CT26 cells (left), and the statistic green intensity (right). Scale bar: 100 μm. **C** MitoSOX images of CT26 cells (left), and the statistic red intensity (right). Scale bar: 100 μm. **D** Flow cytometry images of JC-1 in CT26 cells (left), and the statistic results of JC-1 (right). **E** OX-MitoDNA (8-OHdG) images of CT26 cells (left), and the statistic red intensity (right). Scale bar: 100 μm. n = 3. *P < 0.05, **P < 0.01, ***P < 0.001

Xia et al. discovered that intracellular HAP could undergo biodegradation in an acidic environment and thereby release calcium. To explore this, we used a Fluo-4 AM probe to detect the production of free Ca^2+^ in the cytoplasm of CT26 cells. The results revealed almost no red fluorescence in the control group, whereas weak red fluorescence was observed in the HAP- and HAP@ATO-treated groups, which reflected calcium ion release from HAP into CT26 cells (Fig. 3A). Compared to those in the HAP group, the CS-HAP and CS-HAP@ATO groups showed a dramatic increase in Ca^2+^ in CT26 cells. It is reported that mitochondria are the main targets of Ca^2+^, and overloading Ca^2+^ would induce mitochondrial damage. In addition, ATO could disturb the mitochondrial electron transport chains (ETCs) and thus again cause mitochondrial injury. Therefore, CS-HAP@ATO NPs were supposed to bidirectionally attack mitochondria by causing Ca^2+^ overload and direct mitochondrial damage.

MPTP complexes, situated between mitochondrial inner and outer membranes, play a critical role in maintaining MMP and ion equilibrium between intracellular and extracellular spaces. Illustrated in Fig. 3B, the green fluorescence intensity denoting mitochondrial calcein-AM accumulation via mPTP was found to decrease in response to HAP-associated NPs. This observation signifies augmented mPTP opening within mitochondria, featuring elevated Ca^2+^ levels. Intriguingly, mPTP fluorescence intensities were markedly lower in the HAP@ATO, CS-HAP, and CS-HAP@ATO groups compared to the free HAP group, with CS-HAP@ATO NPs displaying the most pronounced inhibitory effect. The opening of mPTP can induce mitoROS production, MMP depolarization, and heightened mitochondrial injury. To further assess this phenomenon, MitoSOX, a hydroethidine compound targeting mitochondria, was employed to detect mitoROS production. Figure 3C illustrates the scenario: the control group exhibited minimal red fluorescence, HAP-based groups displayed faint red fluorescence, and the CS-HAP@ATO-treated group exhibited notably strong red fluorescence (approximately 4-fold increase), indicating significant intracellular mitoROS generation in the treated cells. Moreover, JC-1 fluorescent probe, a cationic anabolic dye, was applied to gauge changes in mitochondrial MMP. A decrease in red fluorescence or red/green ratio signifies declining MMP. As revealed in Fig. 3D, HAP-treated groups experienced significant reduction in red/green fluorescence ratios compared to the control group. The CS-HAP@ATO-treated group exhibited the most pronounced alterations: substantial red fluorescence attenuation and notable green fluorescence augmentation, with the ratio plummeting to a mere 1/5 of the control group. Due to their vulnerability and limited repair capabilities, mitoDNA is susceptible to mitoROS assault, leading to Ox-mitoDNA formation. We employed 8-OHdG (red) to label Ox-mtDNA in CT26 cells and gauge the impact of HAP-based NPs on mitoDNA damage. As depicted in Fig. 3E, minimal red fluorescence indicative of Ox-mitoDNA was evident in the control group. Conversely, the red fluorescence intensity was approximately 4-fold, 5-fold, 8-fold, and 15-fold higher in the HAP-treated, HAP@ATO-treated, CS-HAP-treated, and CS-HAP@ATO-treated groups, respectively. This underscores the heightened mitoDNA damage in CS-HAP@ATO-treated CT26 cells.

### CS-HAP@ATO NPs activates mitoDNA-pyroptosis pathway

**Fig. 4:**
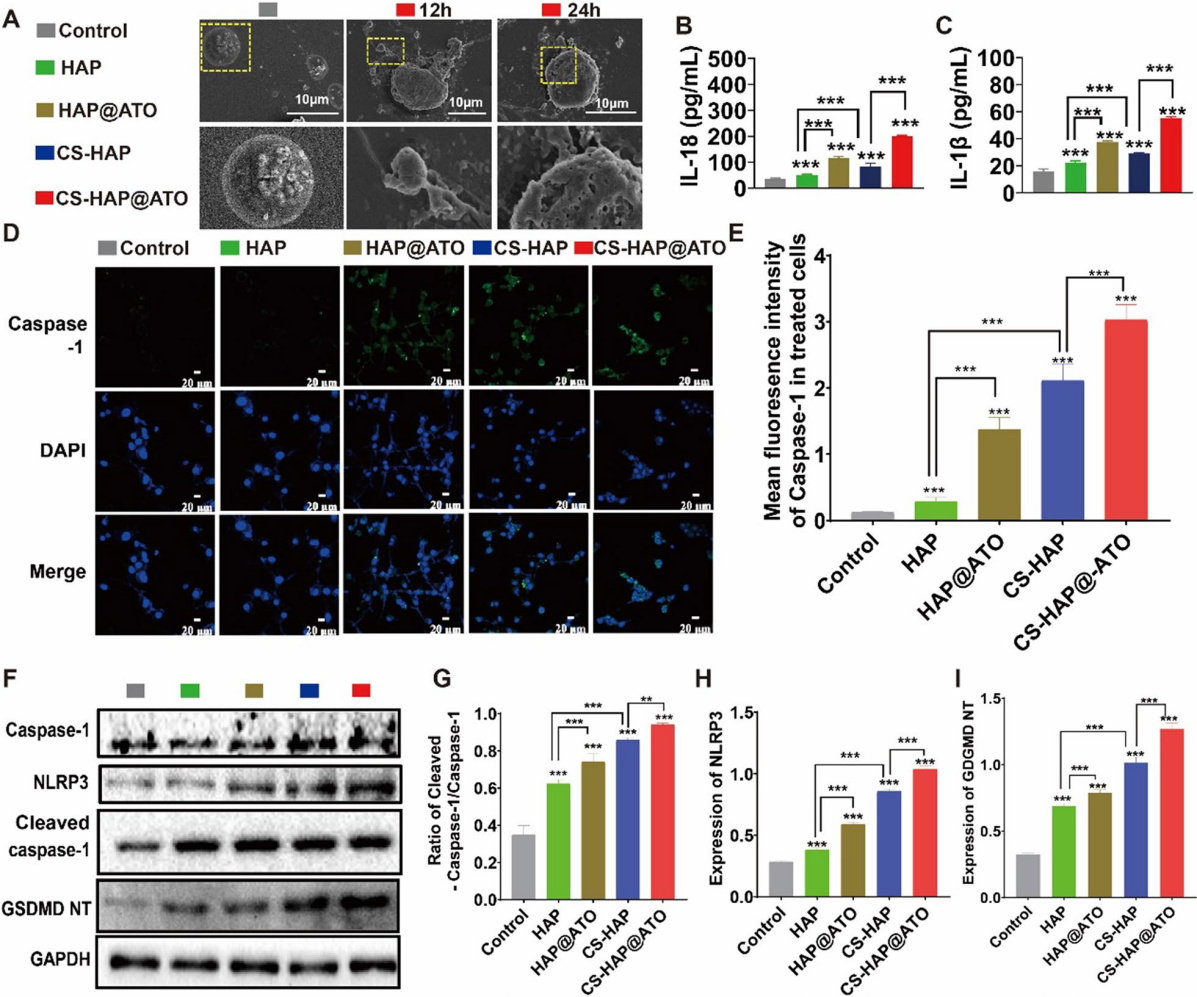
Pyroptosis activation of CS-HAP@ATO NPs. **A** SEM images of CT26 cells after being treated with HAP-based NPs. **B** Secreted immune factor IL-18 inside CT26 cells after being treated with HAP-based NPs. **C** Secreted immune factor of IL-1β inside CT26 cells after being treated with HAP-based NPs. (D&E) CLSM images of caspase-1 (green) inside CT26 cells after being treated with HAP-based NPs (left), and the statistic green intensity (right). Scale bar: 100 μm. **F–I** Western blot results of pyroptosis pathway inside CT26 cells after being treated with HAP-based NPs (left), and the statistic expression of Cleaved Caspase-1, NLRP3, and GSDMD NT proteins, analyzed by Image J (right). n = 3. *P < 0.05, **P < 0.01, ***P < 0.001

Pyroptosis, an inflammatory programmed cell death pathway, plays a vital role in the body’s innate immune defense. According to the classical paradigm, cellular exposure to danger signals activates intracellular pattern recognition receptors like NLRP3. These receptors, in collaboration with adaptor proteins and pro-caspase-1, assemble into inflammasomes. The activation of caspase-1 leads to cleavage and polymerization of GSDMD, provoking cellular swelling and rupture and releasing a plethora of intracellular contents and pro-inflammatory mediators. The initiation of NLRP3 inflammasome activity is a pivotal trigger for pyroptosis. These inflammasomes can react to diverse stimuli, such as mitochondrial oxidative stress and the liberation of tissue proteases/phospholipids. Our preliminary investigations suggested that oxidative damage to mitoDNA effectively prompted NLRP3 activation, thus initiating pyroptosis. In this context, the release of ox-mitoDNA following CS-HAP@ATO NP treatment could potentially instigate the pyroptosis pathway. To validate this hypothesis, we employed SEM to examine the morphology of cells and identify those exhibiting pyroptosis characteristics by their distinct “bubbling” appearance. As shown in Fig. 4A, Additional file 1: Fig. S5, unlike the control group, the cells in each drug treatment group showed different degrees of morphological changes. Notably, the CS-HAP@ATO treatment group exhibited swollen and vacuolated cells, which clearly demonstrated the occurrence of pyroptosis. Correspondingly, enhanced secretion of IL-18 and IL-1β, indicative of the pyroptosis-associated inflammatory response, was observed in HAP-based treated groups (Fig. 4B, C). The heightened green fluorescence observed in cells treated with CS-HAP@ATO NPs among all HAP-induced groups (Fig. 4D, E) denoted efficient caspase-1 activation. Moreover, augmented expression of key pyroptosis-associated proteins, including NLRP3, caspase-1, cleaved caspase-1, and GSDMD, was evident in HAP-based NP-treated cells, with the CS-HAP@ATO group exhibiting the most pronounced effect (Fig. 4F–I). These results provided compelling evidence that specific pyroptosis was triggered via the NLRP3/Caspase-1/GSDMD pathway.

### In vivo anti-tumor efficiency of CS-HAP@ATO NPs

**Fig. 5:**
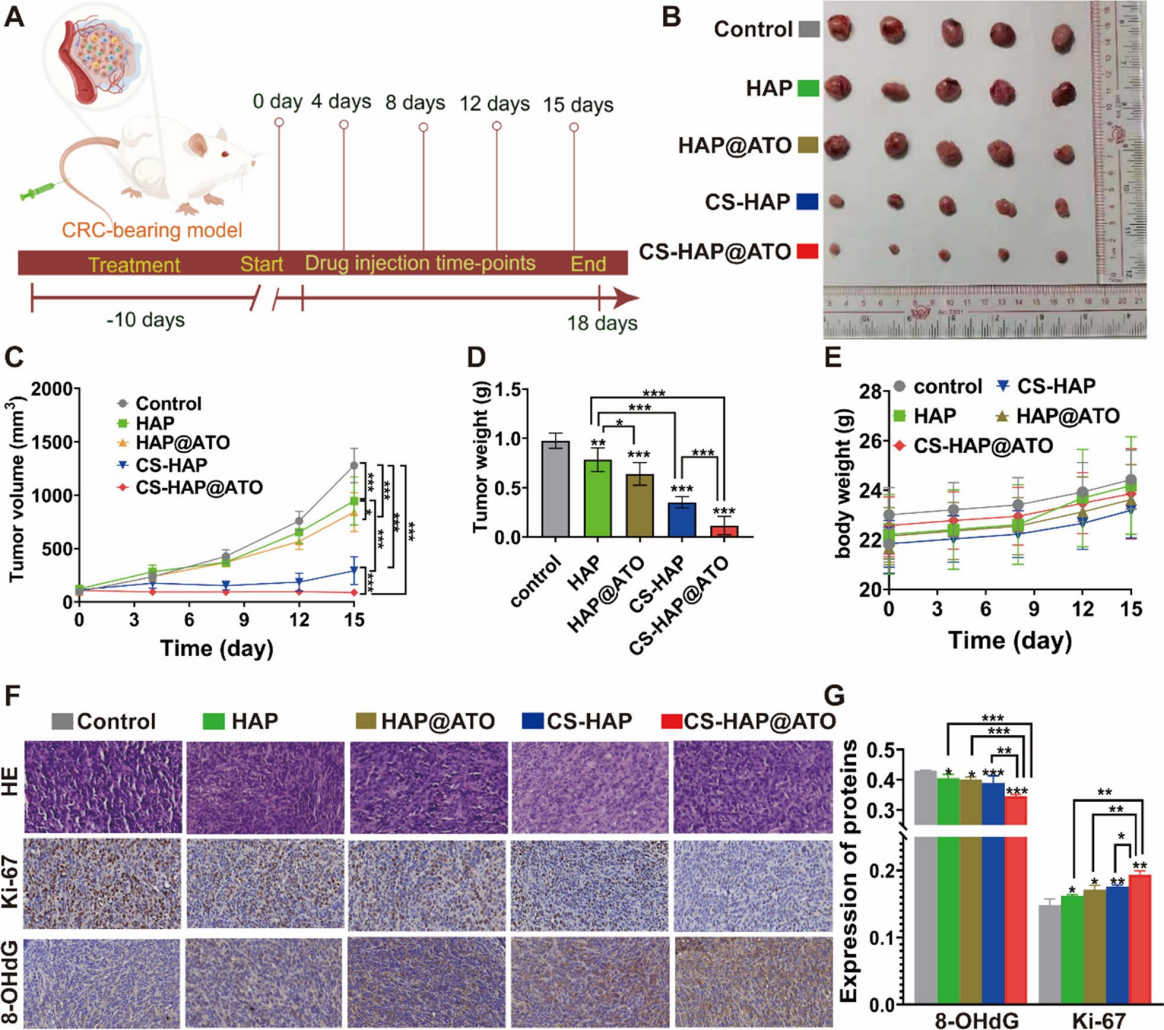
In-vivo antitumor effect of CS-HAP@ATO NPs. **A** Schematic illustration of in vivo injection of CS-HAP@ATO NP for CRC-bearing mice. **B** Diagram of tumor isolated from treated mice. **C**–**E** Tumor volume, tumor weight and body weight of treated mice. **F** HE, Ki-67 and 8-OHdG staining of tumor isolated from treated mice, 400×. **G** The expression of Ki-67 and 8-OHdG analyzed from their staining slices. n = 5. * P  < 0.05, ** P  < 0.01, ***P  < 0.001

Biosafety is a step that must be monitored before drug application. Therefore, hemolysis test were demonstrated to validate their biocompatibility. The findings indicated that there was no obvious hemolysis phenomenon as drug concentration increased, and that the hemolysis rate of all experimental groups was lower than 5% (Additional file 1: Fig. S6). This provided further evidence supporting the safety of CS-HAP NPs. To evaluate the potential therapeutic impact of CS-HAP@ATO NPs in vivo, a subcutaneous tumor model using tumor-bearing mice was established and randomly divided into five groups: control (saline), HAP, HAP@ATO, CS-HAP, and CS-HAP@ATO. Mice were subjected to intravenous injections of the respective drugs over five cycles, culminating in euthanization on the 15th day (Fig. 5A). The obtained results (Fig. 5B, C) illustrated that tumor volumes were significantly reduced in all HAP-based NPs groups, compared to the control group. Among all, CS-HAP@ATO demonstrated the most effective inhibitory rate (93%), though HAP (25%), HAP@ATO (34%), and CS-HAP (70%) also inhibited well anti-tumor effects. Additionally, measurements of tumor weights on the 15th day revealed a marked reduction in the CS-HAP@ATO-treated group (85%) compared to others (Fig. 5D). These outcomes distinctly indicate the notable inhibition of tumor growth through the administration of CS-HAP@ATO NPs, while our results showed no obvious body-weight influence (Fig. 5E). To further affirm the in vivo therapeutic potential, we meticulously examined the tumor tissues’ proliferation and apoptosis status using paraffin-embedded tumor sections. Analysis of tumor cell apoptosis and proliferation was accomplished using H&E, Ki-67 and TUNEL staining (Fig. 5F, G), which demonstrated decreased expression of Ki-67 and upregulated expression of TUNEL in response to HAP-associated NPs, compared to control group. Additionally, CS-HAP@ATO NPs did exert most significant changes in proliferation and apoptosis markers. This robustly indicates that CS-HAP@ATO NPs wield pronounced in vivo antitumor efficacy.

### CS-HAP@ATO NPs remodeling Tumor immune microenvironment (TIME)

**Fig. 6:**
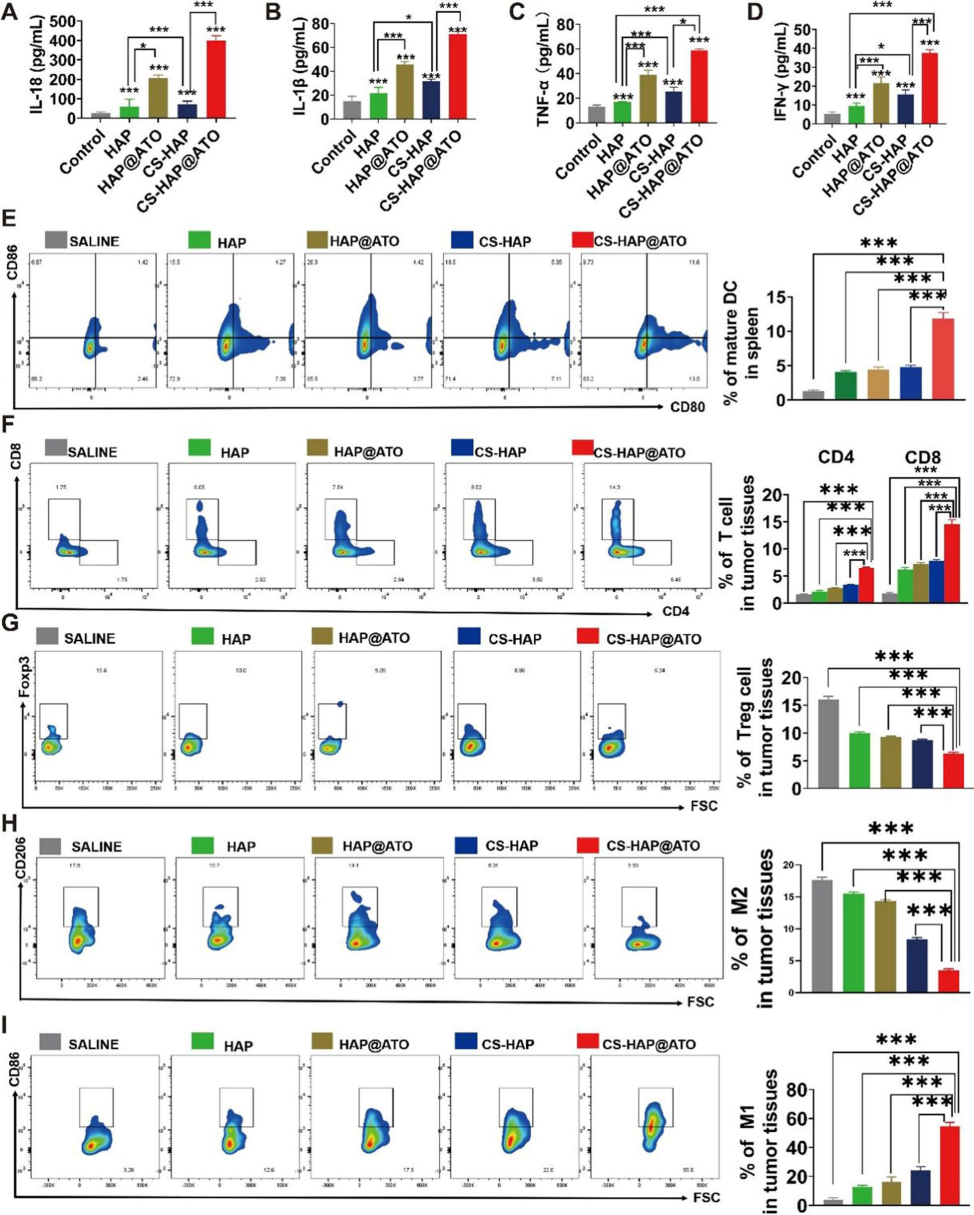
Immune recruitment effect of CS-HAP@ATO NPs. **A**–**D** Elisa assay of pro-inflammatory cytokines, including IL-18, IL-1β, TNF-α and IFN-γ inside tumor issues. **E** Flow cytometry analysis of DCs cells (CD45^+^CD11c^+^CD80^+^CD86^+^) in tumor-draining lymph nodes. **F** CD4^+^ (CD45^+^CD3^+^CD4^+^) and CD8^+^ T cells (CD45^+^CD3^+^CD8^+^) in tumor samples extracted from mice. **G** Treg cells (CD45^+^CD3^+^CD4^+^CD25^+^FOXP3^+^) in tumor samples extracted from mice given treatments. **H** M2 type cells (CD206^+^) in tumor samples extracted from mice. **I** M1 type cells (CD86^+^) in tumor samples extracted from mice. *P < 0.05, **P < 0.01, ***P < 0.001

Tumor immunotherapy has experienced rapid advancements, transforming the landscape of cancer treatment. Nonetheless, the challenge of achieving robust response rates persists as a significant hurdle. In this context, the activation of pyroptosis has emerged as a novel avenue with immense potential. Pyroptosis activation not only initiates an inflammatory response but also triggers a substantial release of inflammatory cytokines. Our ELISA findings (Fig. 6A–D) underscored this by showcasing elevated secretion levels of crucial pro-inflammatory cytokines, encompassing IL-18, IL-1β, TNF-α, and IFN-γ. Accumulating research emphasizes that pyroptosis, as an inflammation-driven cell death process orchestrated by gasdermin, plays a pivotal role in recruiting and activating immune cells, thereby orchestrating an immune-mediated eradication of tumors. This unique interplay between cellular pyroptosis, cancer immunity, and tumor evolution has indeed revolutionized anti-tumor strategies. In line with these insights, our previous data reaffirms the notion that pyroptosis activation triggers the heightened expression and release of inflammation-associated factors. This intricate interplay effectively bolsters the ability of dendritic cells (DCs) to present antigens to T and B cells, orchestrating the initiation of innate immunity. This heightened cytokine expression further accentuates the immune-stimulatory environment, fostering a conducive milieu for tumor clearance.

Dendritic cells (DCs) play a pivotal role in orchestrating the immune response by facilitating the production of type-I interferons and serving as key antigen-presenting cells for both innate and acquired immunity. To probe the potential of CS-HAP@ATO treatment in driving DC maturation and augmenting immune response, we conducted an in-depth assessment through flow cytometry, focusing on markers indicative of DC maturation as well as the expression of CD80 and CD86 on the cell surface. The results presented in Fig. 6E provided compelling evidence that the CS-HAP@ATO treatment notably upregulated the expression of CD80 and CD86 on DCs compared to the control treatment. This significant augmentation underscored the enhancement of DC maturation triggered by CS-HAP@ATO, ultimately fostering a more robust immune response. In the context of antitumor immunity, CD8^+^ T cells are indispensable protagonists. These T lymphocytes wield their potency through the secretion of perforin and granzyme B, playing a pivotal role in fortifying CD8^+^ CTL responses. Conversely, CD4^+^CD25^+^FOXP3^+^ regulatory T (Treg) cells exert immunosuppressive effects, curtailing the efficacy of tumor effector T cells and often contributing to tumor immune evasion. Notably, elevated Treg cell counts have been associated with dismal prognoses across various malignancies. Our comprehensive flow cytometric analysis unveiled a profound landscape shift in the CS-HAP@ATO-treated group. The CS-HAP@ATO treatment led to a marked surge in the presence of CD8^+^ and CD4^+^ cells within the tumor microenvironment, positioning CS-HAP@ATO as an enhancer of immune cell infiltration (Fig. 6F). Moreover, the frequency of Treg cells registered a significant reduction upon CS-HAP@ATO treatment (Fig. 6G), signifying the attenuation of Treg-mediated immunosuppression.

During the initial stages of tumor development, which correspond to the acute inflammatory phase, macrophages can be activated into M1 macrophages by various cytokines such as IFNγ, TNF-α, IL-1, as well as pathogen-associated molecular patterns (PAMPs) like bacterial lipopolysaccharides and damage-associated molecular patterns (DAMPs) like HMGB1. This M1 macrophage activation plays a crucial role in inhibiting tumor growth. However, if the acute inflammatory response fails to subside in a timely manner, it can transition into chronic inflammation, leading to the production of suppressive cytokines that activate M2 macrophages. Consequently, the conversion of M2 macrophages to M1 macrophages becomes a significant approach for reshaping the immune microenvironment and enhancing the responsiveness to tumor immunotherapy. And it is worth noting that the frequency of M1 cells in the CS-HAP@ATO model was significantly lower than that in the control group **(**Fig. 6H), and the frequency of M2 cells was in contract increased (Fig. 6I), demonstrating that CS-HAP@ATO exacerbate the imbalance of M1/M2. Therefore, our results demonstrate that CS-HAP@ATO could efficiently remodel the TIME.

## Conclusions

In summary, we constructed a tumor-targeting nanodrug delivery system (CS-HAP@ATO). These designed NPs exhibit appropriate size distributions, negative surface potentials, and pH-responsive drug release capacities. Through CD44-receptor-mediated endocytosis, CS-HAP@ATO NPs target cells and enhance cellular drug uptake. The internalized NPs release ATO and Ca^2+^, which further alter the MMP, activate the mitoROS/OX-mitoDNA/pyroptosis pathway, accompanied by increased NLRP3 inflammasome, Caspase-1, and GSDMD expression. Since the release of immune inflammatory factors (IL-1β and IL-18), CS-HAP@ATO NPs could remodel the TIME by inducing DC maturation, recruiting CD8^+^ and CD4^+^ T cells, reducing Treg cells and improve macrophage polarization. In conclusion, we achieved effective inhibition of tumor growth through enhanced immunotherapy, which provides a potential clinical application for promoting anti-tumor immunotherapy (Scheme 1).

### Supplementary Information

**Additional file 1: Figure S1. **Thermogravimetric analysis of CS-HAP@ATO NPs. **Figure S2. **The standard curve equations of ATO. **Figure S3. **The standard curve equations of Calcium. **Figure S4. **TEM images of CT26 cells treated with CS-HAP@ATO NPs. **Figure** **S5.**The SEM images of CT26 cells after being treated with HAP-based NPs. **Figure** **S6.** The hemolytic test of CS-HAP@ATO NPs.
