# Supplementary material for: Tumor-targeting hydroxyapatite nanoparticles for remodeling tumor immune microenvironment (TIME) by activating mitoDNA-pyroptosis pathway in cancer
Source: J Nanobiotechnology. 2023 Dec 7;21:470. doi: 10.1186/s12951-023-02231-4 (PMC10704647; doi:10.1186/s12951-023-02231-4)
Supplement: Supplementary file 1 — Additional file 1: Figure S1. Thermogravimetric analysis of CS-HAP@ATO NPs. Figure S2. The standard curve equations of ATO. Figure S3. The standard curve equations of Calcium. Figure S4. TEM images of CT26 cells treated with CS-HAP@ATO NPs. Figure S5.The SEM images of CT26 cells after being treated with HAP-based NPs. Figure S6. The hemolytic test of CS-HAP@ATO NPs. [file 12951_2023_2231_MOESM1_ESM.docx]

**Tumor-targeting hydroxyapatite nanoparticles for remodeling tumor immune microenvironment (TIME) by activating mitoDNA-pyroptosis pathway in cancer**

**Supporting Information**


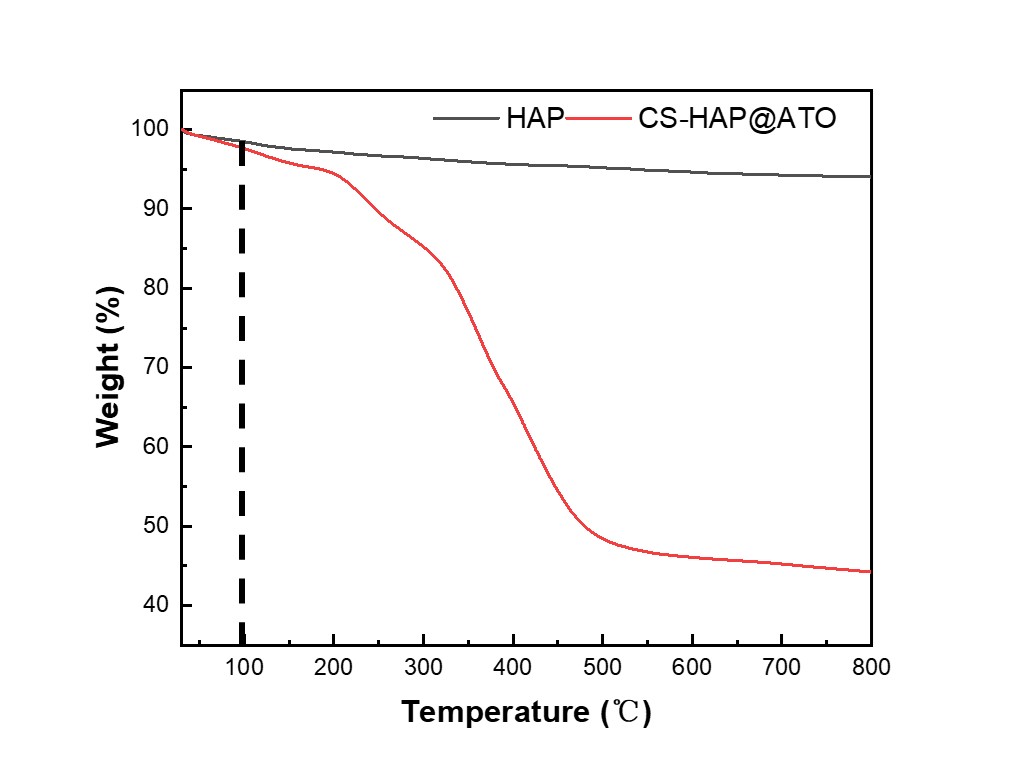


**Figure S1.** Thermogravimetric analysis of CS-HAP@ATO NPs.


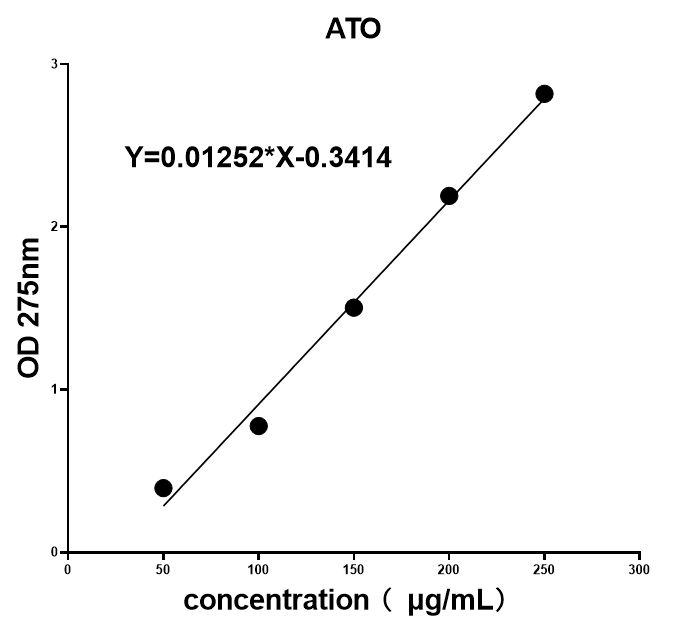


**Figure S2.** The standard curve equations of ATO.


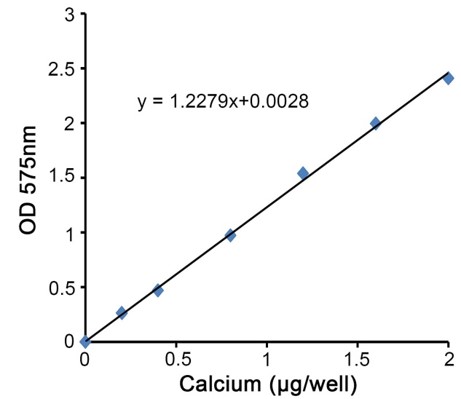


**Figure S3.** The standard curve equations of Calcium.


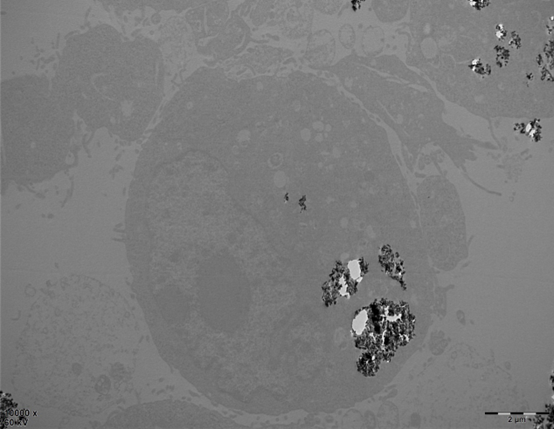


**Figure S4.** TEM images of CT26 cells treated with CS-HAP@ATO NPs.


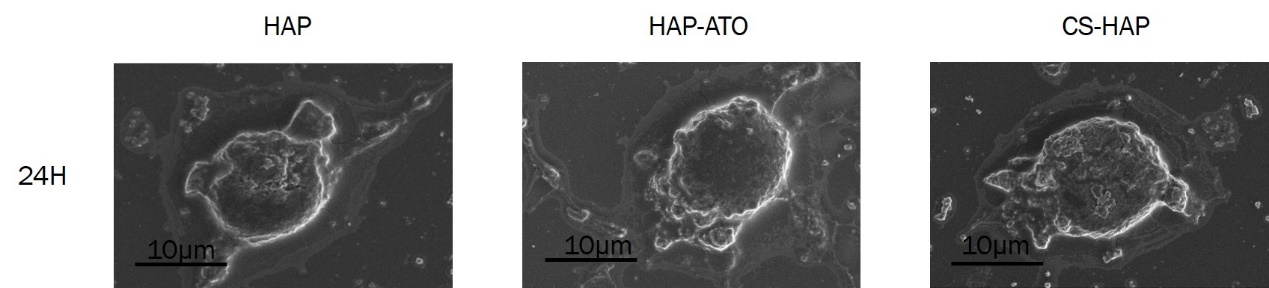


**Figure** **S5.** The SEM images of CT26 cells after being treated with HAP-based NPs.


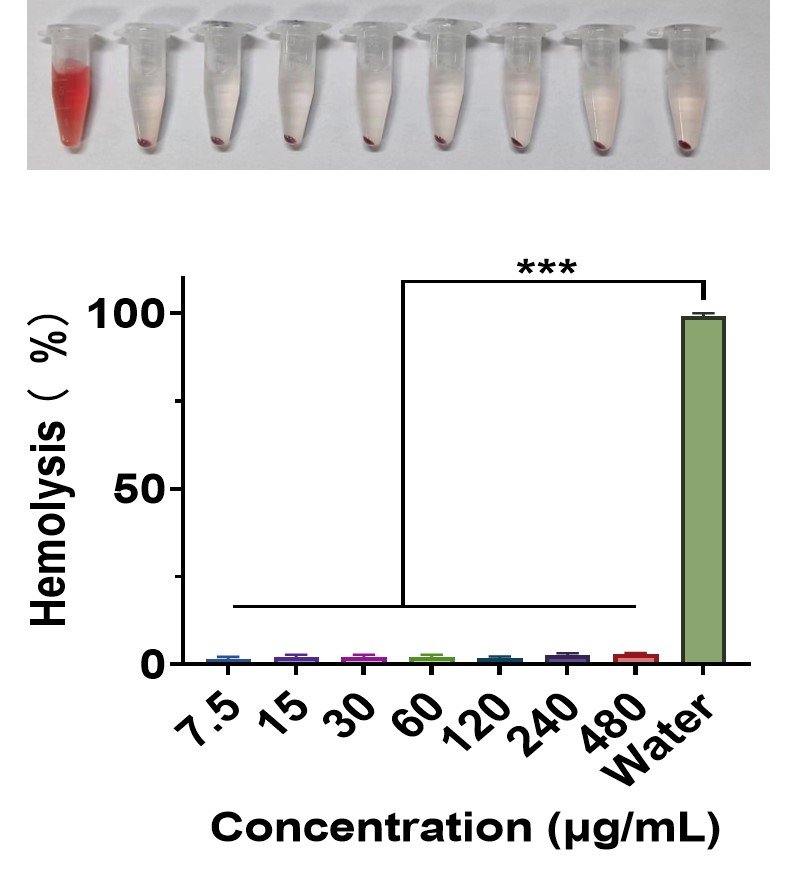


**Figure** **S6.** The hemolytic test of CS-HAP@ATO NPs.
